# An Augmented Sample Selection Framework for Prediction of Anticancer Peptides

**DOI:** 10.3390/molecules28186680

**Published:** 2023-09-18

**Authors:** Huawei Tao, Shuai Shan, Hongliang Fu, Chunhua Zhu, Boye Liu

**Affiliations:** 1 Key Laboratory of Food Information Processing and Control, Ministry of Education, Henan University of Technology, Zhengzhou 450001, China; thw@haut.edu.cn (H.T.); ss@stu.haut.edu.cn (S.S.); jackfu_zz@163.com (H.F.); zhuchunhua@haut.edu.cn (C.Z.); 2 Henan Engineering Laboratory of Grain IOT Technology, Henan University of Technology, Zhengzhou 450001, China; 3 College of Food Science and Engineering, Henan University of Technology, Zhengzhou 450001, China

**Keywords:** anticancer peptides, prediction model, data augmentation, noisy samples, uncertainty estimation, confidence, pseudo-label

## Abstract

Anticancer peptides (ACPs) have promising prospects for cancer treatment. Traditional ACP identification experiments have the limitations of low efficiency and high cost. In recent years, data-driven deep learning techniques have shown significant potential for ACP prediction. However, data-driven prediction models rely heavily on extensive training data. Furthermore, the current publicly accessible ACP dataset is limited in size, leading to inadequate model generalization. While data augmentation effectively expands dataset size, existing techniques for augmenting ACP data often generate noisy samples, adversely affecting prediction performance. Therefore, this paper proposes a novel augmented sample selection framework for the prediction of anticancer peptides (ACPs-ASSF). First, the prediction model is trained using raw data. Then, the augmented samples generated using the data augmentation technique are fed into the trained model to compute pseudo-labels and estimate the uncertainty of the model prediction. Finally, samples with low uncertainty, high confidence, and pseudo-labels consistent with the original labels are selected and incorporated into the training set to retrain the model. The evaluation results for the ACP240 and ACP740 datasets show that ACPs-ASSF achieved accuracy improvements of up to 5.41% and 5.68%, respectively, compared to the traditional data augmentation method.

## 1. Introduction

One of the greatest challenges in health is cancer. It is the second leading cause of death worldwide, accounting for approximately one in six deaths [1,2,3]. Despite the limited successes in some cases, conventional anticancer therapies still have many problems. For example, immunotherapy shows a low efficacy of 10% to 30% [4]. Radiotherapy leads to non-negligible side effects, such as radiation osteonecrosis, fibrosis, cognitive impairment, and nerve damage [5]. Chemotherapy introduces chemicals into the body to attack cancer cells, the long-term use of which increases drug resistance, and the likelihood of recurrence is extremely high [6]. Furthermore, chemotherapeutic drugs indiscriminately kill normal and cancerous cells [7].

Anticancer peptides (ACPs) are biologically active peptides with anti-tumor activity. The discovery of ACPs has provided a new perspective on cancer therapy, and they have been extensively studied [8,9,10]. ACPs are cationic in nature, allowing them to selectively kill cancer cells by interacting with their anionic cell membrane components [11]. ACPs are highly selective, highly penetrative, easy to chemically modify, and have broad-spectrum anticancer activity and low production costs [12,13]. Compared to conventional drugs, ACPs are safer, produce lower drug resistance, and thus, have become a competitive therapeutic option. Therapies based on ACPs have been widely explored at different stages of preclinical and clinical trials against various tumor types [14,15,16]. However, traditional experiments for ACP identification are usually time-consuming and costly, limiting the discovery and development of ACPs. Hence, efficient prediction techniques are urgently needed to expedite and facilitate this process.

Artificial intelligence techniques can save time and reduce costs in identifying candidate ACPs. Earlier researchers used classical machine learning methods for ACP prediction. Hajisharifi et al. [17] used PseAAC to represent peptide sequences and applied locally aligned kernels as precomputed kernels in support vector machines (SVM) for ACP prediction. Chen et al. [18] optimized the g-gap dipeptide composition and developed a sequence-based predictor. Manavalan et al. [19] developed a predictor for ACPs based on SVM and random forests. Furthermore, Rao et al. [11] proposed a prediction method that fuses multi-view information. Boopathi et al. [20] developed a prediction model called mACPred by inputting the prediction probability obtained from the optimal model based on feature encoding as a feature vector into SVM.

In recent years, data-driven deep learning techniques have demonstrated great potential for predicting ACPs. Wu et al. [21] proposed a prediction model based on the Word2vec [22] word embedding method and convolutional neural networks (CNNs). To fully utilize the peptide sequence information, Yi et al. [23] combined k-mer sparse matrix features with binary features, which were then fed into a long short-term memory (LSTM) neural network for prediction. Meanwhile, Yu et al. [24] compared the performances of three network architectures, CNN, recurrent neural network (RNN), and CNN-RNN, for ACP prediction, and the results showed that the RNN achieved the highest performance. Lv et al. [25] applied transfer learning to past work, using two pre-trained models for peptide sequences in feature extraction, and proposed a prediction tool for ACPs called iACP-DRLF. Then, Akbar et al. [26] used a word embedding strategy based on FastText [27] to represent peptide sequences for ACP prediction. Yuan et al. [28] proposed an integrated model combining Bi-LSTM, CNN, and machine learning algorithms. Zhou et al. [29] designed a model called TriNet. Three parallel networks with different structures were used in this model to process sequence fingerprints, sequence evolutions, and physicochemical properties of peptides. In the study by Yao et al. [30], a flexible and easy-to-train deep forest [31] structure was used as a prediction model, and FEGS [32]-encoded peptide sequences were utilized. These works provide a powerful background for predicting peptide sequences with anticancer activity.

However, ACP prediction remains challenging. Data-driven computational methods require many samples to train models, and data scarcity can result in inadequate generalization capabilities. Unfortunately, the publicly available ACP dataset in [33] is limited to 970 ACP samples. The two publicly available datasets in [23] contain 376 and 129 ACP samples, respectively. By comparison, two widely used image classification datasets, Cifar-10 [34] and ImageNet [35], contain 60,000 and 14 million images, respectively. As a result of the dataset size limitation, the model may not thoroughly learn the features and patterns of such a small number of ACP samples.

The augmentation of data is a method of solving the limited data problem, which has proven effective in other tasks, such as computer vision [36], natural language processing [37], and speech recognition [38]. Chen et al. [39] used data augmentation in ACP prediction for the first time by proposing a model called ACP-DA, which adds perturbations to the samples in the feature space to generate new samples. Bhattarai et al. [40] investigated a similar data augmentation approach. It should be noted, however, that this traditional data augmentation method may not apply to all situations. According to [28], after implementing this data augmentation method, model performance decreased. A similar phenomenon was reported in another study [41]. This may be caused by the introduction of noisy samples during the data augmentation process, which results in the model learning incorrect information. Unfortunately, as far as we know, there is rarely a particularly effective data augmentation method in the field of predicting anticancer peptides. Therefore, constructing an effective data augmentation method has become an urgent problem to be solved in ACP prediction under limited sample conditions.

**Figure 1:**
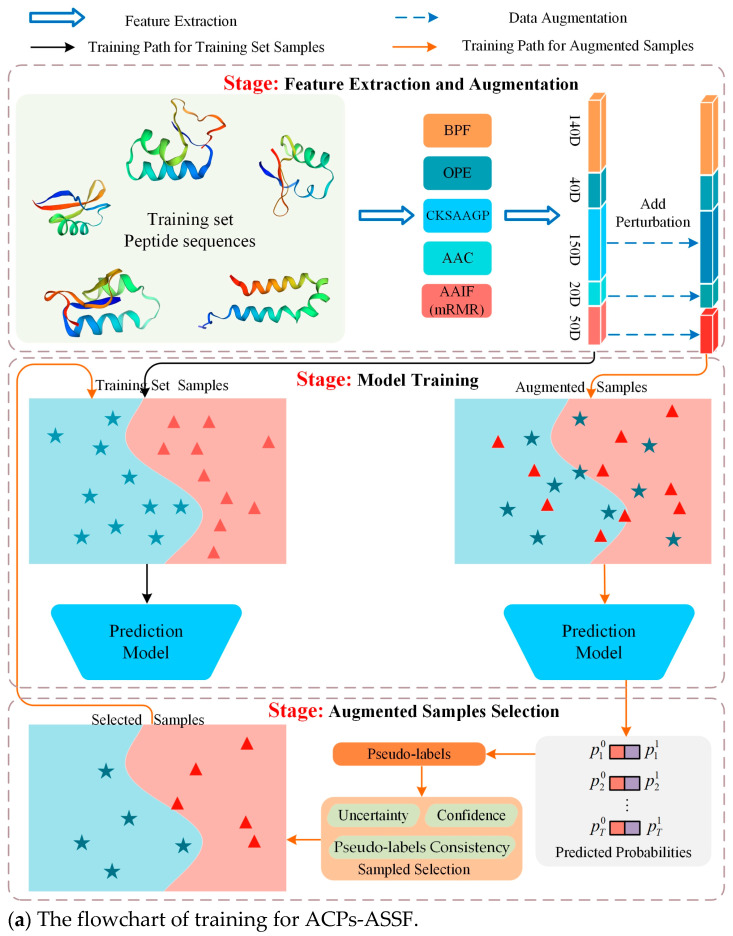

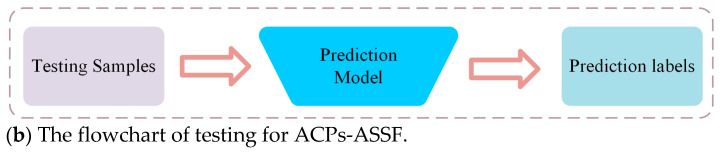
Flowchart of ACPs-ASSF. Peptide sequences were encoded as 400-dimensional feature vectors by five types of peptide descriptors, and perturbations were added to the feature vectors to generate augmented samples.

Therefore, in this work, we propose an augmented-sample selection framework, called ACPs-ASSF, for the prediction of anticancer peptides to ensure the controllability of data augmentation by excluding noisy samples. ACPs-ASSF is capable of selecting high-quality augmented samples for automatic training. A flowchart of the proposed method is shown in Figure 1. First, the features are extracted from peptide sequences and augmented samples are obtained by adding perturbations. Then, the prediction model is trained by training set samples, the augmented samples are fed into the prediction model, pseudo-labels are computed, and the model prediction uncertainty is estimated. Augmented samples with low uncertainty, high confidence, and pseudo-labels consistent with the original labels are selected, and the selected samples are merged into the training set to retrain the model. Several iterations of the above process are performed to ensure that the samples chosen will be useful for model training. Finally, the testing samples are input into the trained prediction model, and the prediction labels are obtained.

The main contributions of this article are as follows.

1. This article constructs a novel augmented sample selection framework that can effectively remove noisy samples from the augmented samples, thereby ensuring the performance of the model.
2. This article constructs a pseudo-label screening mechanism based on uncertainty, confidence, and label consistency, which can ensure the quality of augmented samples after screening.

## 2. Results

### 2.1. Performance Evaluation Metrics

We followed the widely used evaluation metrics [42,43,44,45,46,47] to evaluate the performance of the proposed method, including its accuracy, specificity, F1-score, and Matthews correlation coefficient (MCC), as defined below:

(1) $$\mathrm{Accuracy}=\frac{TN+TP}{TN+TP+FN+FP}$$

(2) $$\mathrm{Specificity}=\frac{TN}{TN+FP}$$

(3) $$F1-\mathrm{score}=\frac{2TP}{2TP+FP+FN}$$

(4) $$\mathrm{MCC}=\frac{TP*TN-FP*FN}{\sqrt{\left(TP+FP)(TP+FN)(TN+FP)(TN+FN\right)}}$$

where TN represents the true negative number, TP represents the true positive number, FN represents the false negative number, and FP represents the false positive number.

### 2.2. Comparison of ACPs-ASSF with Other Methods

The performance of ACPs-ASSF and the traditional data augmentation method was evaluated with the ACP240 and ACP740 datasets [23]. In addition, we report the baseline method’s performance. The three methods are described below.

Baseline: A prediction model trained without data enhancement.

Traditional data augmentation (TDA) [39,40]: A prediction model trained using all augmented samples.

ACPs-ASSF: The method proposed in this paper.

To ensure a fair comparison, the same prediction model was used for all three methods, and the training-related hyperparameter settings were kept the same.

#### 2.2.1. Performance Comparison under Different Numbers of Augmented Samples

It is important to note that there are two main parameters that affect the performance of a model when using data augmentation. One is N/R, which controls the number of augmented samples, indicating the ratio of the number of new samples generated to the number of original samples. The other parameter is ω, the perturbation factor, which controls the magnitude of the added perturbation values. We first compared the performance of the ACPS-ASSF and TDA methods under different N/R conditions. Table 1 and Table 2 show the comparison results on the ACP240 and ACP740 datasets.

Using ACP240, compared to the baseline, TDA showed very limited performance improvement with different N/R settings and even decreased accuracy, specificity, and MCC metrics in many cases. The ACPs-ASSF achieved improvements in all metrics under different N/R settings. Compared to the baseline, the maximum improvements in accuracy, specificity, F1-score, and MCC were 3.75%, 3.63%, 3.83%, and 6.95%, respectively.

Using ACP740, the TDA method performed worse. Compared to the baseline, TDA decreased in all four metrics with different N/R settings. In contrast, ACPs-ASSF achieved improvements in all metrics under different N/R settings; the maximum improvements in accuracy, specificity, F1-score, and MCC were 4.46%, 6.44%, 4.13%, and 8.97%, respectively, in ACPs-ASSF compared to the baseline.

#### 2.2.2. Performance Comparison under Different Perturbation Factors

We then compared the performance of the ACPs-ASSF with that of the TDA method under various perturbation factors ω. Table 3 and Table 4 show the comparison results for the ACP240 and ACP740 datasets.

As seen in ACP240, the four evaluation metrics of the TDA method reached their highest value when ω=0.003. Nevertheless, specificity decreased by 2.53% compared to the baseline. ACPs-ASSF performed better than the baseline for all metrics under five different ω settings. When ω=0.005, ACPs-ASSF showed superior overall performance, with the accuracy, F1-score, and MCC reaching, respectively, 80.00%, 81.07%, and 60.32%, which were 4.58%, 4.34%, and 9.11% higher than the baseline, respectively.

In ACP740, the TDA metrics were lower than those of the baseline under all five ω settings, except for ω=0.004, where the specificity of TDA was slightly higher than that of the baseline. The evaluation metrics of ACPs-ASSF under all five ω settings were higher than those for the baseline and TDA. With ω=0.005, the ACPs-ASSF showed significant performance improvements, with the accuracy, F1-score, and MCC reaching 76.22%, 76.66%, and 52.34%, respectively, which were 3.11%, 2.64%, and 6.14% higher than the baseline.

As shown above, ACPs-ASSF significantly outperformed TDA in both datasets, with different numbers of augmented samples and different perturbation factors. TDA improved the performance of a few evaluation metrics in very few cases. In contrast, ACPs-ASSF demonstrated a remarkable performance improvement under different parameter settings. Furthermore, we analyzed the noisy samples that were excluded from the ACPs-ASSF. We found that, in ACP240, the number of noisy samples excluded by the ACPs-ASSF was 55.26% of the number of all augmented samples. Among these noisy samples, 57.09% were positive samples and 42.91% were negative samples. In ACP740, the number of noisy samples excluded by the ACPs-ASSF was 14.70% of the number of all augmented samples. Among these noisy samples, 62.76% were positive samples and 37.24% were negative samples.

### 2.3. Visualization of ACPs-ASSF Selected Samples

**Figure 2:**
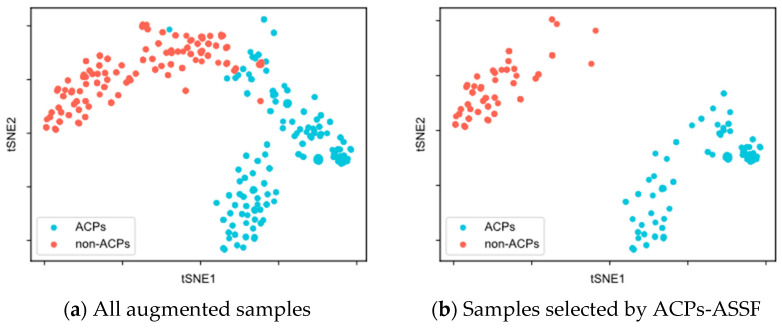
t-SNE visualization of the samples in the ACP240 dataset.

**Figure 3:**
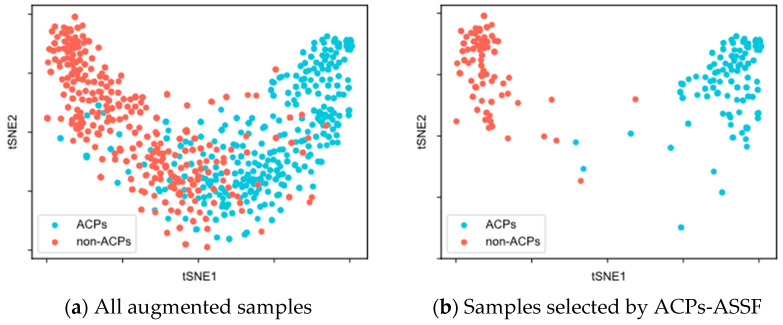
t-SNE visualization of the samples in the ACP740 dataset.

To evaluate the quality of the augmented samples selected by ACPs-ASSF, we performed t-SNE [48] visualization of the augmented data, as shown in Figure 2 and Figure 3.

Figure 2a demonstrates the distribution of all augmented samples in ACP240. The augmented samples formed two class clusters, with ACPs mainly distributed in the lower right of the plane, non-ACPs mainly in the upper left, and a region of overlap in the middle. As shown in Figure 2b, after ACPs-ASSF selection, noisy samples in the middle of the two class clusters, as well as those outside the class cluster distribution, were eliminated, and the selected samples exhibited a larger inter-class distribution distance.

Figure 3a illustrates the distribution of all augmented samples for ACP740. There was a greater overlap between the distributions of the two classes, along with insufficient compactness within each class. In Figure 3b, the two distinct class clusters from the ACPs-ASSF selection and the number of samples outside the distribution of class clusters decrease.

### 2.4. Hyperparametric Sensitive Verification of ACPs-ASSF

Since ACPs-ASSF introduces new hyperparameters, including an uncertainty threshold λ, confidence threshold γ, and number of stochastic forward pass times T, we analyze their impact on ACPs-ASSF performance in this section.

**Figure 4:**
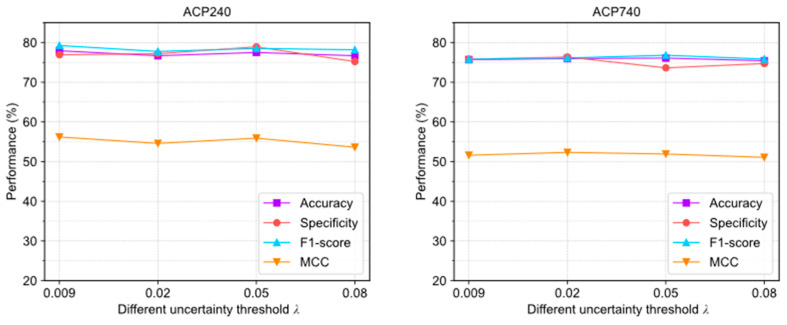
The performance changes of ACPs-ASSF on the ACP240 and ACP740 datasets under different uncertainty threshold λ settings.

Figure 4 illustrates the evaluation results of ACPs-ASSF under four different λ settings. In the ACP240 and ACP740 datasets, the trends of the changes in accuracy, specificity, F1-score, and MCC are relatively stable; this indicates that the algorithm is not highly sensitive to the parameters λ.

**Figure 5:**
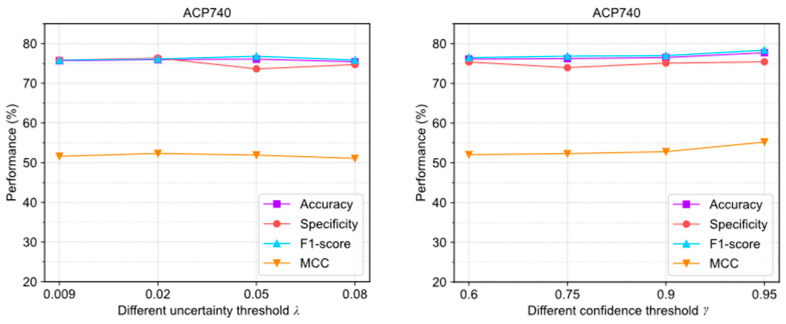
The performance changes of ACPs-ASSF on ACP240 and ACP740 datasets under different confidence threshold γ settings.

As shown in Figure 5, the evaluation results were obtained with different γ settings. The extreme deviations of the four evaluation metrics were 0.84%, 1.71%, 1.33%, and 1.78%, which were all within 2%. In ACP740, they were 1.62%, 1.48%, 1.89%, and 3.17%, respectively. This algorithm is not highly sensitive to the parameters γ either.

**Figure 6:**
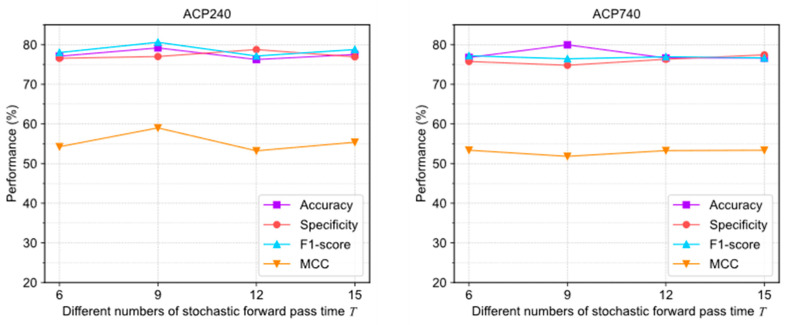
The performance changes of ACPs-ASSF on ACP240 and ACP740 datasets under different numbers of stochastic forward pass time T settings.

Figure 6 illustrates the evaluation results under different T settings. In ACP240, the extreme deviations of the four evaluation metrics were 2.92%, 2.2%, 3.46%, and 4.75%, respectively. In ACP740, the extreme deviations were 3.33%, 1.52%, 0.76%, and 1.55%, respectively. The curves of various indicators fluctuate significantly, indicating that the algorithm is more sensitive to pass times T.

The above results show that adjusting the uncertainty threshold λ and confidence threshold γ does not cause significant performance differences in ACPs-ASSF, which indicates that the method is robust to the above hyperparameter values. However, algorithm performance is sensitive to the number of iterations.

### 2.5. Performance of Various Feature Combinations

**Figure 7:**
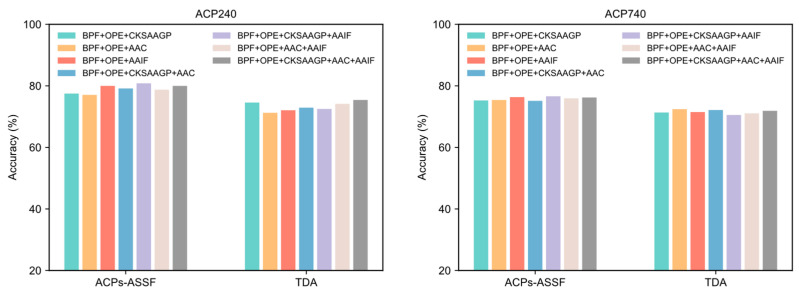
The performance of ACPs-ASSF and TDA on the ACP240 and ACP740 datasets of various feature combinations.

The results reported previously in this paper were generated by representing peptide sequences using the concatenation of five different peptide descriptors. In this section, we evaluate the performance of other feature encoding combinations. The performance achieved by each feature encoding combination in both datasets is shown in Figure 7. The accuracy obtained by ACPs-ASSF was significantly higher than that of TDA under different feature combinations.

## 3. Discussion

For ACP prediction, traditional data augmentation fails to steadily improve the model’s predictive performance. It has been reported in the literature [28,41] that the model’s performance decreases when data augmentation is used. This may be due to the generation of noisy samples during data augmentation. To address this issue, we propose a framework for augmented sample selection named ACPs-ASSF. In ACPs-ASSF, we utilize the uncertainty of the model prediction to guide the sample selection process to filter out high-quality augmented samples. Using ACPs-ASSF can reduce the impact of noisy samples generated during data augmentation on model performance, thus resulting in improved performance.

We compared ACPs-ASSF with the traditional data augmentation method from two perspectives: the number of generated samples and the perturbation factor. Using the same prediction model and training-related hyperparameters, ACPs-ASSF exhibited superior performance over the traditional data augmentation and baseline methods. The traditional data augmentation method provides minimal enhancements to model performance. The performance is even lower than the baseline method in many cases, a phenomenon consistent with previous studies [28,41]. Thus, we conclude that noisy samples impact the model’s prediction performance. ACPs-ASSF can prevent the model from learning incorrect information caused by noisy samples during training, thus steadily improving performance. By visualizing the samples, we found that the augmented samples had low inter-class separation, which was more evident in ACP740, where the sample distributions overlap more. The model can be confused by samples in the overlapped region as well as those outside the class clusters. Although some samples within the class clusters are also screened out, this is acceptable, as the selected samples show higher interclass separations and smaller intraclass distances. This suggests that ACPs-ASSF picks more discriminative samples favorable for model training. Then, we analyzed the hyperparameters introduced for ACPs-ASSF. The evaluation results of ACPs-ASSF in the two datasets varied slightly under different settings. Therefore, ACPs-ASSF is not sensitive to the uncertainty threshold λ and confidence threshold γ and only sensitive to pass times T. This enables it to maintain reliable performance under various experimental conditions. Finally, we evaluated the performance under different feature combinations. The results show that the accuracy of ACPs-ASSF was significantly higher than that of TDA under the same feature combinations, which further proves the effectiveness of the proposed ACPs-ASSF.

Despite the apparent advantages of ACPs-ASSF, it can be further improved. Although the samples selected by ACPs-ASSF demonstrated strong discrimination, there were also a few samples that were beneficial for model training that were not selected. Therefore, in future work, we will endeavor to retrain as many samples as possible while eliminating noisy samples. By using more high-quality samples to train the model, the prediction performance will be further improved.

## 4. Materials and Methods

### 4.1. Datasets

To demonstrate that the proposed method is effective, it was evaluated on two benchmark datasets, ACP240 and ACP740 [23]. In these two datasets, peptide sequences with more than 90% similarity were removed. ACPs that have been experimentally validated are considered positive sequences, whereas antimicrobial peptides without an anticancer function are considered negative sequences. There are 740 sequences in ACP740, of which 376 are positive and 364 are negative. There are 240 sequences in ACP240, of which 129 are positive and 111 are negative. There is no overlap between ACP740 and ACP240. The label corresponding to each sequence displays whether the sequence is positive or negative.

### 4.2. Feature Extraction

Each peptide sequence is encoded by the following five types of peptide descriptors: binary profile feature (BPF), ordinal positional encoding (OPE), composition of K-Spaced amino acid group pairs (CKSAAGP), amino acid composition (AAC), AAindex feature (AAIF). Each descriptor is specified below.

#### 4.2.1. BPF

In the standard amino acid alphabet, there are 20 different amino acids (i.e., A, C, D, E, F, G, H, I, K, L, M, N, P, Q, R, S, T, V, W, and Y). For each amino acid type, it is represented using a 20-dimensional one-hot vector. For example, the first amino acid type A in the alphabet is represented by ${\left(1,\;0,\;0,\;\ldots ,\;0\right)}_{20}$. The second amino acid, type C, is represented by ${\left(0,\;1,\;0,\;\ldots ,\;0\right)}_{20}$. According to Yi et al. [23], we selected only the first seven amino acids in each peptide sequence for coding. As a result, each sample was encoded as a 140-dimensional vector via BPF.

#### 4.2.2. OPE

OPE was introduced by Yuan et al. [28]. Since OPE can only encode peptide sequences with a fixed length, pre-processing was required. The length of peptide sequences was standardized to 40 when extracting this feature. For peptide sequences longer than 40, excess amino acids were removed. Peptide sequences less than 40 in length were supplemented by “X”. When extracting OPE, integers from 0 to 19 were used to represent the different amino acid types, and integers from 0 to 39 were used to represent the position of the amino acid in the peptide sequence. If the currently encoded amino acid type was m (0≤m≤19), which is the l (0≤l≤39) amino acid in the peptide sequence, then the amino acid was encoded as m∗40+l. “X” was coded as “−1”. Each sample was encoded as a 40-dimensional vector via OPE.

#### 4.2.3. CKSAAGP

The 20 amino acid residues were divided into 5 groups, including the aliphatic residue group (G1: GAVLMI), the aromatic residue group (G2: FYW), the positively charged residue group (G3: KRH), the negatively charged residue group (G4: DE), and the uncharged residue group (G5: STCPNQ). CKSAAGP calculates the frequency of amino acid group pairs separated by any k residues. If k=0, there are 25 0-spaced amino acid group pairs (i.e., G1G1, G1G2, …, G5G5), and the feature vector can be defined as:

(5) $$k=0,\;{\left(\frac{N_{G1G1}}{L-(k+1)},\;\frac{N_{G1G2}}{L-(k+1)},\;\ldots ,\;\frac{N_{G5G5}}{L-(k+1)}\right)}_{25}$$

where L is the peptide sequence length and the number of times the group pair G1G1 appears in the peptide sequence. In this paper, we jointly considered k=0, 1, 2, 3, 4, 5. A 150-dimensional feature vector was obtained through CKSAAGP.

#### 4.2.4. AAC

AAC calculates the frequency of occurrence of the 20 standard amino acids in a given peptide sequence, generating a 20-dimensional vector. The feature vector obtained by AAC encoding can be defined as:

(6) $${\left(\frac{N_{A}}{L},\;\frac{N_{C}}{L},\;\ldots ,\;\frac{N_{Y}}{L}\right)}_{20}$$

where $N_{A}$ represents the number of occurrences of amino acid type A in the peptide sequence.

#### 4.2.5. AAIF

The length of peptide sequences is also standardized to 40 when extracting AAIF features. We used the iFeature [49] tool to extract the AAIF. Peptide sequences with a length of 40 produce AAIF vectors with a dimension of 21,240, which is excessively high and results in a dimension disaster. In order to reduce dimensionality, we used the mRMR [50] feature selection algorithm, and the final AAIF feature dimension was 50.

The dimensions of the vectors obtained by the above encoding methods were 140, 40, 150, 20, and 50, respectively. Finally, these five features were connected to form a 400-dimensional feature vector used to represent the peptide sequence.

### 4.3. Data Augmentation

In traditional data augmentation, new samples are generated by adding perturbation values to the feature vector [39,40]. In this paper, only CKSAAGP, AAC, and AAIF were added with perturbations since BPF and OPE are not suitable for adding perturbations. The new augmentation samples obtained can be defined by the following equation:

(7) $$x_{a}=x_{o}*V*\omega +x_{o}$$

where $x_{o}$ is a randomly selected sample from the training set. V is a 400-dimensional vector consisting of two parts. The first part is a 180-dimensional zero vector corresponding to the BPF and OPE features to ensure that no perturbations are added to these two parts. The second part is a 220-dimensional random vector with values uniformly distributed between 0 and 1. ω is the perturbation coefficients, which are used to control the magnitude of the added perturbation values.

### 4.4. Pseudo-Labeling and Uncertainty Estimation

In this paper, pseudo-labels [51] were determined by models trained on the original data, and hard labels were chosen as pseudo-labels for the augmented samples. Assuming that $p^{c}$ represents the probability value predicted by the model that the augmented sample $x_{a}$ belongs to class c, the pseudo-label of $x_{a}$ obtained by the traditional method can be calculated by Equation (8).

(8) $$y^{\prime }=\arg\max_{c}p^{c}$$

Thus, the class with the largest probability value was chosen as the pseudo-label for $x_{a}$. However, poor calibration of the neural network may result in incorrect predictions with a high degree of confidence [52]. For this reason, we considered uncertainty estimation when selecting the pseudo-labels. We used the standard deviation of the model outputs to quantify the uncertainty. However, because the model’s parameters are fixed, the standard deviation cannot be calculated from a single output value. This means that the learned model parameters must follow a distribution rather than being fixed values. The model parameters can be sampled from this distribution, and each sampling results in a different set of model parameters, which changes the model output. This can be achieved by the dropout method in deep neural networks. Dropout refers to the drop of some neurons with probability p during the forward pass of a neural network. As a result, when a model is trained using dropout, its parameters can be thought of as following a Bernoulli distribution. In practice, we kept dropout on during the model prediction phase and performed T stochastic forward passes. The prediction uncertainty was obtained by calculating the standard deviation from the T outputs of the model, as shown in Equation (9).

(9) $$u(p^{c})=\sqrt{\frac{1}{T-1}\sum_{t=1}^{T}{\left(p_{t}^{c}-{\bar{p}}^{c}\right)}^{2}}$$

This method of estimating uncertainty is called Monte Carlo dropout [53]. Then, the calculation of the pseudo-labeling of $x_{a}$ becomes Equation (10).

(10) $$y^{\prime }=\arg\max_{c}(\frac{1}{T}\sum_{t=1}^{T}p_{t}^{c})$$

### 4.5. ACPs-ASSF

The original training set consisting of N samples is denoted as $D_{o}={\left\{(x_{i},\;y_{i})\right\}}_{i=1}^{N}$, where $x_{i}$ is the feature vector and $y_{i}$ is the label of $x_{i}$. Data augmentation is implemented on $D_{o}$ to obtain the augmented dataset $D_{a}={\left\{(x_{j},\;y_{j})\right\}}_{j=1}^{M}$. Using $D_{s}$ to denote the dataset consisting of the selected augmented samples, it can be defined by the following equation:

(11) $$D_{s}={\left\{(x_{j},\;y_{j})|(u(p_{j}^{c})<\lambda )\cap (\mu (p_{j}^{c})>\gamma )\cap (y_{j}^{\prime }=y_{j})\right\}}_{j=1}^{H}$$

where $\mu (p^{c})=\frac{1}{T}\sum_{t=1}^{T}p_{t}^{c}$ is the confidence of the model prediction, λ is the uncertainty threshold, γ is the confidence threshold, H<M, and $D_{s}\subset D_{a}$.

The training procedure of ACPs-ASSF is summarized in Algorithm 1. In practice, the loss function for training the model is cross-entropy (CE) and the optimization algorithm is the stochastic gradient descent (SGD). In each iteration, the parameters of the prediction model are initialized.
**Algorithm 1** Uncertainty-aware augmented sample selection (ACPs-ASSF)**Require:** Original training dataset $D_{o}$; augmented sample dataset $D_{a}$; prediction model $f_{\theta }$ with trainable parameters θ; uncertainty threshold λ and confidence threshold γ; number of stochastic forward pass times T; number of iterations for selecting samples I; number of epochs E for training model.1:$D=D_{o}$; ▷ obtain training set2: **for** iteration=1 **to** I **do**
3:       Initialize θ;
4:       **if** iteration>1
5:           $D=D_{o}\cup D_{s}$;▷ merge the selected samples to the training set6:       **for** epoch=1 **to** E **do**
7:             Train $f_{\theta }$ using D; ▷ using CE loss and SGD8:       **end for**

9:       **for** 
t=1
 **to** 
T
 **do**

10:              Dropout(θ);
11:              Input samples from $D_{a}$ into $f_{\theta }$; ▷ accumulate the output of each pass12:        **end for**

13:        Compute the uncertainty and pseudo-labels by Equations (9) and (10);14:        Use Equation (11) to obtain $D_{s}$; ▷ select augmented samples15: **end for**

16: **return** θ


### 4.6. Experimental Settings

In this paper, all experimental results were obtained using a five-fold cross-validation strategy. The prediction model contains five fully connected layers, which were set as ((400, 256), (256, 64), (64, 32), (32, 8), and (8, 2)). The activation function was Relu. After the first and second layers, dropout was implemented with a dropout rate of 0.3. The learning rate was tuned dynamically, and the initial learning rate was 0.001. The batch sizes of ACP240 and ACP740 were 32 and 64, respectively. The number of iterations for selecting samples I was 10 and the number of epochs for the training model E was 50. As a default, the uncertainty threshold λ was set to 0.03, the confidence threshold γ was set to 0.8, and the number of stochastic forward pass times T was set to 10.

## 5. Conclusions

For ACP prediction, the existing data augmentation method generates noisy samples, leading to incorrect model learning and, thus, reduced performance. In this paper, we address this problem by proposing ACPs-ASSF, an augmented sample selection framework, for improving the performance of ACP prediction. ACPs-ASSF guides the selection of samples based on the uncertainty of model predictions. The evaluation results for the ACP240 and ACP740 datasets demonstrate the potential of ACPS-ASSF in improving the prediction of ACPs. The visualization of the augmented samples demonstrates that the selected samples have excellent inter-class separation, which facilitates the model training. In addition, ACPs-ASSF is a generalized framework that does not require any specific model architecture and can be easily combined with other prediction methods to enhance performance. Consequently, ACPs-ASSF is expected to become a competitive tool in the field of ACP prediction to facilitate the high-throughput screening of ACPs.

## Figures and Tables

**Table 1:** The performance comparison of TDA and ACPs-ASSF on ACP240 under five different N/R settings, with ω fixed at 0.006 (the best metrics are in bold).

Methods	N/R	Accuracy	Specificity	F1-Score	MCC
Baseline	-	75.42	76.08	76.73	51.21
TDA	1	76.25	71.55	78.41	52.86
	2	75.42	68.97	77.94	51.69
	3	73.75	68.74	76.01	47.58
	4	73.33	65.58	76.36	47.46
	5	75.42	69.85	77.78	51.36
ACPs-ASSF	1	78.75	78.91	79.95	57.97
	2	77.08	**79.71**	77.75	54.98
	3	77.92	79.15	78.54	55.85
	4	77.92	77.55	79.02	55.65
	5	**79.17**	77.67	**80.56**	**58.16**

**Table 2:** The performance comparison of TDA and ACPs-ASSF on ACP740 under five different N/R settings, with ω fixed at 0.006 (the best metrics are in bold).

Methods	N/R	Accuracy	Specificity	F1-Score	MCC
Baseline	-	73.11	70.13	74.02	46.20
TDA	1	72.30	69.05	73.22	44.55
	2	71.89	69.10	72.69	44.00
	3	71.35	66.99	72.82	42.95
	4	72.03	69.47	73.01	44.17
	5	71.89	68.40	73.04	43.99
ACPs-ASSF	1	75.95	75.37	76.39	52.18
	2	76.22	73.95	76.84	52.31
	3	75.68	75.19	75.92	51.32
	4	77.43	74.86	**78.15**	54.77
	5	**77.57**	**76.57**	77.90	**55.17**

**Table 3:** The performance comparison of TDA and ACPs-ASSF on ACP240 under five different ω settings, with N/R fixed at 2 (the best metrics are highlighted in bold).

Methods	ω	Accuracy	Specificity	F1-Score	MCC
Baseline	-	75.42	76.08	76.73	51.21
TDA	0.001	75.83	70.00	78.21	52.43
	0.002	75.42	70.51	77.51	51.08
	0.003	77.08	73.55	78.92	54.83
	0.004	71.67	67.86	73.84	43.93
	0.005	75.42	69.08	77.86	51.72
ACPs-ASSF	0.001	77.50	76.59	79.07	56.25
	0.002	77.50	**80.71**	78.19	55.91
	0.003	78.33	79.08	79.60	57.59
	0.004	77.08	77.11	78.35	54.88
	0.005	**80.00**	78.88	**81.07**	**60.32**

**Table 4:** The performance comparison of TDA and ACPs-ASSF on ACP740 under five different ω settings, with N/R fixed at 2 (the best metrics are highlighted in bold).

Methods	ω	Accuracy	Specificity	F1-Score	MCC
Baseline	-	73.11	70.13	74.02	46.20
TDA	0.001	72.16	69.69	72.95	44.33
	0.002	71.35	68.61	72.36	42.80
	0.003	72.03	69.91	72.74	44.21
	0.004	71.89	70.66	72.41	43.63
	0.005	71.89	69.58	72.54	43.84
ACPs-ASSF	0.001	75.81	74.34	76.31	51.42
	0.002	75.14	74.04	75.56	50.25
	0.003	75.81	76.30	75.84	51.77
	0.004	76.08	**76.50**	76.04	52.16
	0.005	**76.22**	75.14	**76.66**	**52.34**

## Data Availability

Publicly available datasets were analyzed in this study. The datasets (ACP240 and ACP740) can be found at: https://github.com/haichengyi/ACP-DL (accessed on 8 August 2023).
